# Desert Dust Outbreaks in Southern Europe: Contribution to Daily PM_10_ Concentrations and Short-Term Associations with Mortality and Hospital Admissions

**DOI:** 10.1289/ehp.1409164

**Published:** 2015-07-24

**Authors:** Massimo Stafoggia, Stefano Zauli-Sajani, Jorge Pey, Evangelia Samoli, Ester Alessandrini, Xavier Basagaña, Achille Cernigliaro, Monica Chiusolo, Moreno Demaria, Julio Díaz, Annunziata Faustini, Klea Katsouyanni, Apostolos G. Kelessis, Cristina Linares, Stefano Marchesi, Sylvia Medina, Paolo Pandolfi, Noemí Pérez, Xavier Querol, Giorgia Randi, Andrea Ranzi, Aurelio Tobias, Francesco Forastiere, the MED-PARTICLES Study Group

**Affiliations:** 1 Department of Epidemiology, Lazio Regional Health Service, Rome, Italy; 2 Regional Centre for Environment and Health, Regional Agency for Environmental Prevention of Emilia-Romagna, Modena, Italy; 3 Laboratory of Environmental Chemistry, Aix Marseille Université, Marseille, France; 4 Department of Hygiene, Epidemiology and Medical Statistics, University of Athens Medical School, Athens, Greece; 5 Centre for Research in Environmental Epidemiology (CREAL), Barcelona, Spain; 6 Universitat Pompeu Fabra (UPF), Barcelona, Spain; 7 CIBER Epidemiología y Salud Pública (CIBERESP), Barcelona, Spain; 8 Health Authority Sicily Region, Palermo, Italy; 9 Department of Epidemiology and Environmental Health, Regional Environmental Protection Agency of Piedmont, Turin, Italy; 10 National School of Public Health, Carlos III Health Institute, Madrid, Spain; 11 Environmental Department, Municipality of Thessaloniki, Thessaloniki, Greece; 12 Environmental Health Department, French Institute for Public Health Surveillance, Saint-Maurice, France; 13 Department of Public Health, Bologna Local Health Authority, Bologna, Italy; 14 Institute of Environmental Assessment and Water Research, Barcelona, Spain; 15 Department of Clinical Sciences and Community Health, Università degli Studi di Milano, Milan, Italy

## Abstract

**Background:**

Evidence on the association between short-term exposure to desert dust and health outcomes is controversial.

**Objectives:**

We aimed to estimate the short-term effects of particulate matter ≤ 10 μm (PM10) on mortality and hospital admissions in 13 Southern European cities, distinguishing between PM10 originating from the desert and from other sources.

**Methods:**

We identified desert dust advection days in multiple Mediterranean areas for 2001–2010 by combining modeling tools, back-trajectories, and satellite data. For each advection day, we estimated PM10 concentrations originating from desert, and computed PM10 from other sources by difference. We fitted city-specific Poisson regression models to estimate the association between PM from different sources (desert and non-desert) and daily mortality and emergency hospitalizations. Finally, we pooled city-specific results in a random-effects meta-analysis.

**Results:**

On average, 15% of days were affected by desert dust at ground level (desert PM10 > 0 μg/m3). Most episodes occurred in spring–summer, with increasing gradient of both frequency and intensity north–south and west–east of the Mediterranean basin. We found significant associations of both PM10 concentrations with mortality. Increases of 10 μg/m3 in non-desert and desert PM10 (lag 0–1 days) were associated with increases in natural mortality of 0.55% (95% CI: 0.24, 0.87%) and 0.65% (95% CI: 0.24, 1.06%), respectively. Similar associations were estimated for cardio-respiratory mortality and hospital admissions.

**Conclusions:**

PM10 originating from the desert was positively associated with mortality and hospitalizations in Southern Europe. Policy measures should aim at reducing population exposure to anthropogenic airborne particles even in areas with large contribution from desert dust advections.

**Citation:**

Stafoggia M, Zauli-Sajani S, Pey J, Samoli E, Alessandrini E, Basagaña X, Cernigliaro A, Chiusolo M, Demaria M, Díaz J, Faustini A, Katsouyanni K, Kelessis AG, Linares C, Marchesi S, Medina S, Pandolfi P, Pérez N, Querol X, Randi G, Ranzi A, Tobias A, Forastiere F, MED-PARTICLES Study Group. 2016. Desert dust outbreaks in Southern Europe: contribution to daily PM10 concentrations and short-term associations with mortality and hospital admissions. Environ Health Perspect 124:413–419; http://dx.doi.org/10.1289/ehp.1409164

## Introduction

The International Panel on Climate Change (IPCC) has estimated that most of the atmospheric particles worldwide are emitted by natural sources, with mineral dust from arid regions being the second after marine aerosol (IPCC 2007). The main source areas of dust particles are located in the arid regions of the planet, and Sahara and the Arabian Peninsula have been identified as the major contributors over the globe (Prospero et al. 2002). The Mediterranean basin is especially vulnerable to injections of desert dust because of its proximity to Sahara to the south and to the Arabian Peninsula to the east. Over the Mediterranean, desert dust outbreaks are caused by certain meteorological scenarios, well characterized for western, central, and eastern sides of the basin (Escudero et al. 2005; Gkikas et al. 2013; Kalivitis et al. 2007; Querol et al. 2009b; Salvador et al. 2014).

Short-term effects of particulate matter (PM) have been widely documented in the epidemiological literature (Pope and Dockery 2006). The fine fraction of PM (generally identified in the size range ≤ 2.5 μm; PM_2.5_) has long been considered the one mostly responsible for adverse health effects, because it can easily reach the lower respiratory system and influence the circulatory system (Pope and Dockery 2006). Most of the research is now focusing on the major components and sources of PM, including organic constituents and metals originating from traffic emissions and other combustion processes. However, a debate on the potential health effects of coarse particles (PM in the range 2.5–10 μm; PM_2.5–10_) has been stimulated by a systematic review from Brunekreef and Forsberg (2005), where the authors concluded that coarse and fine PM exert similar effects on respiratory outcomes, and supported the plausibility of an association between PM_2.5–10_ and cardiovascular outcomes. Among the major constituents of coarse PM, crustal materials, re-suspended dust, sea salts, desert dust, and biogenic components have been identified (Perrino et al. 2009; Pope and Dockery 2006; Querol et al. 2009b). However, doubt remains on the relative toxicity of these components and, in particular, on whether natural sources of PM represent a threat to human health. Several studies have been conducted in the last few years in single cities of Southern Europe, all evaluating whether the short-term association between PM and mortality/hospitalizations would be enhanced on days characterized by desert dust advections, with inconsistent results (Karanasiou et al. 2012).

We have previously shown results of the associations between short-term exposure to both fine and coarse particles with mortality (Samoli et al. 2013, 2014) and hospital admissions (Stafoggia et al. 2013) in multiple cities of South Europe. The aim of the present study is to describe the geographic distribution of PM_10_ (≤ 10 μm) concentrations from desert dust and other sources in the Euro-Mediterranean area, and report estimates of their short-term effects on mortality and emergency hospitalizations. The work was conducted within the European Union (EU)–funded project “Particles size and composition in Mediterranean countries: geographical variability and short term health effects” (MED-PARTICLES; http://95.110.213.190/medparticles/index.php?lang=en).

## Methods

*Setting.* The study was conducted for the period 2001–2010 in 13 European cities of the Mediterranean basin: Barcelona and Madrid (Spain), Marseille (France), Bologna, Milan, Modena, Palermo, Parma, Reggio Emilia, Rome, and Turin (Italy), Athens and Thessaloniki (Greece). Modena, Parma, and Reggio Emilia have been analyzed altogether as a single conurbation called “Emilia-Romagna” because they are very close and share common environmental and sociodemographic characteristics. The Mediterranean area is characterized by specific characteristics, such as highly urbanized areas in which traffic congestion occurs regularly, elevated sea traffic due to tourism and shipping activities over the Mediterranean Sea, and enhanced formation and accumulation of atmospheric pollutants as a result of diverse meteorological and topographical causes (Querol et al. 2009a). In addition, the entire area is frequently affected by outflows from North African deserts, especially from February through October, but with different seasonal incidences western to eastern across the region (Pey et al. 2013).

*Health data.* For each city, we collected information on daily counts of death from natural (*International Classification of Diseases, 9th* or *10th Revision*–ICD-9/ICD-10 codes: 1–799/A00–R99), cardiovascular (ICD-9/ICD-10 codes: 390–459/I00–I99), and respiratory causes (ICD-9/ICD-10 codes: 460–519/J00–J99). Similarly, we retrieved information on hospital emergency admissions from the hospital discharge databases of each country, as previously described (Stafoggia et al. 2013). For each hospitalization we collected information on the primary diagnosis and the age of the patient, and considered three study outcomes defined on the same ICD codes as those used for mortality: cardiovascular and respiratory admissions for patients ≥ 15 years old and respiratory admissions in the age group 0–14 years. Because data were anonymous and collected as daily counts, no informed consent was needed, and the study was exempted from formal institutional review.

*Exposures.* Daily concentrations of PM_10_, PM_2.5_, and PM_2.5–10_ were retrieved in each city of the study from the regional Environmental Protection Agencies (EPAs). Further details are reported by Stafoggia et al. (2013). Urban background monitoring stations were given priority because they better represent average exposure of the population. When they were missing, they were replaced with traffic monitor, as long as the station was not directly influenced by proximal major roads. When data from multiple monitors were available, they were averaged and missing data were imputed using standard procedures, as described by Samoli et al. (2013) and Stafoggia et al. (2013).

Desert dust advection days were identified in each study area using a combination of tools, including meteorological products [NCEP/NCAR (National Centers for Environmental Predictions, and National Center for Atmospheric Research, NCEP/NCAR Reanalysis Project; http://www.esrl.noaa.gov/psd/data/reanalysis/reanalysis.shtml)], aerosol maps [BSC-DREAM (Barcelona Supercomputing Center-Dust REgional Atmospheric Model; http://www.bsc.es/earth-sciences/mineral-dust-forecast-system/bsc-dream8b-forecast); NAAPS-NRL (Navy Aerosol Analysis and Prediction System-Naval Research Laboratory; http://www.nrlmry.navy.mil/aerosol/)]; SKIRON (SKIRON dust operational model; http://forecast.uoa.gr/dustinfo.php), air masses back-trajectories [HYSPLIT (Hybrid Single Particle Lagrangian Integrated Trajectory; http://ready.arl.noaa.gov/HYSPLIT.php], and satellite images [Sea-WiFS (Sea-viewing Wide Field-of-view Sensor; http://oceancolor.gsfc.nasa.gov/SeaWiFS/BACKGROUND/)]. Further details are reported by Pey et al. (2013). Once dust outbreaks were detected in each study area, African dust contributions were quantified for every single day in the outbreak by applying the reference EU method (Escudero et al. 2007). This validated procedure computes the expected PM_10_ concentrations at rural monitoring sites in the absence of African dust advection by using 30-day moving 40th percentile to the PM_10_ data series not containing those days affected by African dust. This results in the estimated background PM, whereas the Saharan dust contribution (desert PM) is estimated as the difference between the observed PM_10_ and the estimated background PM (Escudero et al. 2007; Pey et al. 2013). Daily PM_10_ concentrations from desert dust within the city were attributed from the closest rural background site, as described above, under the assumption that the contribution in the city would be the same as that estimated at the rural site, given the wide spatial distribution of African dust advections. Finally, we computed the amount of daily PM_10_ concentrations due to other sources (non-desert PM_10_) by subtracting the desert PM_10_ component from the total concentrations. Unfortunately, the same approach could not be applied on PM_2.5_ and PM_2.5–10_ because the PM_2.5_ rural monitoring network did not operate daily for multiple years in all locations. Therefore, daily concentrations of fine and coarse PM were used only for descriptive purposes.

*Other data.* Other environmental and time trend variables were collected. They include daily ozone concentrations (from EPAs), air temperature (from the closest airport if available, or city monitors otherwise), influenza epidemics (estimated from national surveillance systems, if available, or an index based on respiratory mortality; otherwise based on daily counts of influenza admissions), public holidays (a two-level variable identifying national holidays), and summer population decrease (a three-level variable assuming value 2 in the 2-week period around mid-August, value 1 from mid-July to end of August, except for the aforementioned 2-week period, and value 0 otherwise). See Samoli et al. (2013) and Stafoggia et al. (2013) for additional details.

*Statistical analysis.* The analyses were carried out in two stages, with city-specific analysis in the first stage followed by random-effects multivariate meta-analysis.

In each city, we fitted over-dispersed Poisson regression models, where the daily counts of cause-specific mortality/hospitalization were connected with daily mean PM_10_ concentrations while adjusting for potential confounders. The list of confounding variables was chosen *a priori* and was the same in each city. It included time trends, air temperature, public holidays, summer population decrease, and influenza epidemics. Long-term and seasonal time trends were adjusted for by introducing a three-way interaction term among year, month, and day of the week. This method has been demonstrated to be equivalent to a case-crossover design using a “time-stratified” approach to select control days (Levy et al. 2001; Lu and Zeger 2007; Maclure 1991). We adjusted for temperature by using separate natural splines for high temperatures (lag 0–1) and low temperatures (lag 1–6), as described elsewhere (Stafoggia et al. 2013). This method is very flexible in capturing potential delayed and nonlinear effects of high and low temperatures on health end points—an issue very relevant in the present study because of the strong seasonality of health outcomes, PM exposures, and dust advections. We did not adjust for humidity or other meteorological parameters because the evidence on their short-term health effects is weak and inconsistent (Katsouyanni et al. 2009). Finally, public holidays, summer population decreases, and influenza epidemics were modeled using indicator variables.

Once the adjustment model was defined, we added the exposure term(s). We estimated associations between the study outcomes and total PM_10_ using single-pollutant models. In addition, we used two-pollutant models that included both desert and non-desert PM_10_ concentrations to estimate independent associations with source-specific PM_10_ exposures. The lag structure between PM_10_ and mortality/hospitalization was chosen *a priori* based on previous results in the same cities (Samoli et al. 2013; Stafoggia et al. 2013): The average exposure of the current and previous day (lag 0–1) was used in relation to natural mortality and cardiovascular admissions, and the average of the preceding 6 days exposure (lag 0–5) was used for cardiorespiratory mortality and respiratory hospital admissions.

Additional analyses were performed. First, we evaluated effect modification by season, defined as cold (October–March) and warm (April–September). This analysis was motivated by previous results showing higher associations between PM exposures and health outcomes in the warmer months, and by desert dust advections having a strong seasonality in terms of both occurrence and intensity of the episodes, with different patterns western to eastern along the Mediterranean basin (Pey et al. 2013). We assessed effect modification by stratifying the source-specific PM models by season. Second, we investigated the concentration–response functions between desert and non-desert PM_10_ and natural mortality by fitting, for each city, a natural spline model for the exposures with two equally spaced inner knots, and pooling the city-specific estimates using a meta-smoothing approach (Schwartz and Zanobetti 2000). As a final check, we evaluated whether the estimated main effect of PM_10_ was modified during desert dust days using an interaction term between the desert dust indicator (yes/no) and total PM_10_ concentration.

Third, we tested whether desert dust days were associated with increased mortality and hospitalizations as an independent risk factor, by adding a dichotomous exposure variable (desert dust, yes/no) in the regression model.

In the second stage of the analysis, we pooled the city-specific results using random-effects multivariate meta-analytical procedures according to the method proposed by Jackson et al. (2010), to account for within-city covariance of effect estimates from the two-pollutant models. We tested heterogeneity among the city-specific results by applying the chi-square test from Cochran’s *Q* statistic, and estimated the amount of heterogeneity by computing the *I* ^2^ statistic (Higgins and Thompson 2002), which represents the proportion of total variation in effect estimates due to between-cities heterogeneity. All results are expressed as percent increases in mortality/hospitalizations, with 95% confidence intervals (CI), relative to 10-μg/m^3^ increments in the pollutants. All first-stage analyses were fit using R, version 3.0.2 (R Core Team 2011). Meta-analyses were conducted using Stata, version 11 (StataCorp, College Station, TX, USA).

## Results

*Exposures.* The annual frequency of dust advection days with dust impacting at ground level (desert PM_10_ > 0 μg/m^3^) was 15% across the study areas and ranged between 28.5% in Palermo and 8.8% in Emilia-Romagna, with a clear increasing trend from north to south and toward the center of the Mediterranean basin (Table 1). We found differences in the seasonality of Saharan dust outbreaks across locations, with spring/summer peaks in the western cities and similar frequencies in colder and warmer months in Greece (see Supplemental Material, Figure S1). During desert dust days, mean total PM_10_ concentrations ranged from 54 μg/m^3^ in Athens and Thessaloniki to 33.1 μg/m^3^ in Marseille, with desert PM_10_ concentrations amounting to 25–40% of the total PM_10_ (see Supplemental Material, Table S1). In general, coarse PM concentrations were higher in southern cities during dust days, but no clear geographical pattern is discernible with regard to fine PM. In addition, total PM_10_ concentrations were usually higher on desert dust days than on other days, with a more pronounced difference in southern cities compared with northern cities. City-specific correlations between the environmental variables are reported in Supplemental Material, Table S2. Table 2 displays the estimated differences between desert dust advection days and no-dust days in terms of daily mean PM, ozone, and air temperature, based on a multivariate linear regression model adjusted for month. Both city-specific and pooled results are reported. Once seasonality was taken into account, we found a sharp increase in PM during desert dust days, with PM_10_ variations ranging from 8.8 μg/m^3^ in Rome to 21.7 μg/m^3^ in Athens. The greatest differences between dust days and other days were found for fine particles in Madrid and Barcelona, whereas coarse particles seemed to be more affected by dust advection in Madrid and Athens. Overall, predicted particulate concentrations increased by 13.4 ± 1.5, 6.9 ± 0.9, and 6.2 ± 1.4 μg/m^3^ on desert dust days for total PM_10_, PM_2.5_, and PM_2.5–10_, respectively. Ozone concentrations were similar during desert dust advections and other days (estimated mean difference, –1.8 ± 1.8 μg/m^3^), but air temperature was 2.2 ± 0.2°C higher on dust days after seasonality adjustment (Table 2).

**Table 1:** Occurrence of desert dust days in the 11 cities of the MED-PARTICLES project [*n* (%)].

City^*a*^	Study period	Days without desert dust^*b*^	Days with desert dust^*b*^	Total days
Milan	2007–2010	1,277 (87.4)	171 (11.7)	1,461 (100.0)
Turin	2006–2010	1,612 (88.3)	199 (10.9)	1,826 (100.0)
Emilia-Romagna^*c*^	2008–2010	988 (90.1)	96 (8.8)	1,096 (100.0)
Bologna	2006–2010	1,578 (86.4)	203 (11.1)	1,826 (100.0)
Marseille	2006–2008	882 (80.5)	195 (17.8)	1,096 (100.0)
Rome	2005–2010	1,809 (82.6)	360 (16.4)	2,191 (100.0)
Barcelona	2003–2010	2,518 (86.2)	365 (12.5)	2,922 (100.0)
Thessaloniki	2007–2009	898 (81.9)	110 (10.0)	1,096 (100.0)
Madrid	2001–2009	2,714 (82.6)	446 (13.6)	3,287 (100.0)
Palermo	2006–2009	976 (66.8)	417 (28.5)	1,462 (100.0)
Athens	2007–2009	775 (70.7)	282 (25.7)	1,096 (100.0)
^***a***^Ordered by latitude, north to south. ^***b***^Days with desert dust are defined as days with desert dust advection and estimated PM_10_ concentrations at ground level > 0 μg/m^3^. Values for days with and without desert dust do not add to the total number of days because of days with missing data on PM_10_ concentrations at the rural monitoring site. ^***c***^Emilia-Romagna includes the cities of Parma, Reggio Emilia, and Modena.				

**Table 2:** Estimated differences (Δ ± SE)*^a^* between desert dust advection days and no-dust days*^b^* in terms of daily mean PM_10_ (μg/m^3^), PM_2.5_ (μg/m^3^), PM_2.5–10_ (μg/m^3^), ozone (μg/m^3^), and air temperature (°C): city-specific and pooled results from a random-effects meta-analysis.

City^*c*^	PM_10_	PM_2.5_	PM_2.5–10_	O_3_	Air temperature
Milan	10.0 ± 2.0	6.1 ± 1.7	2.7 ± 0.8	–2.7 ± 1.8	1.9 ± 0.3
Turin	11.6 ± 2.4	8.7 ± 1.7	—	–0.6 ± 1.9	1.4 ± 0.2
Emilia-Romagna^*d*^	11.0 ± 2.7	5.1 ± 1.3	5.8 ± 0.6	2.5 ± 2.2	2.5 ± 0.3
Bologna	12.3 ± 1.4	7.3 ± 1.2	—	–1.7 ± 1.9	2.2 ± 0.2
Marseille	9.4 ± 0.8	4.6 ± 1.0	2.7 ± 0.7	11.4 ± 1.6	2.2 ± 0.2
Rome	8.8 ± 0.8	2.8 ± 0.6	5.9 ± 0.4	–2.8 ± 1.1	2.5 ± 0.2
Barcelona	13.8 ± 0.9	9.7 ± 0.6	4.2 ± 0.6	–1.7 ± 1.1	2.1 ± 0.2
Thessaloniki	10.0 ± 1.8	6.6 ± 1.1	3.9 ± 0.9	–13.6 ± 1.9	1.2 ± 0.3
Madrid	21.2 ± 1.0	9.8 ± 0.7	11.6 ± 0.8	–2.1 ± 0.7	3.2 ± 0.2
Palermo	16.7 ± 0.9	—	—	–6.1 ± 0.8	2.1 ± 0.1
Athens	21.7 ± 1.3	8.4 ± 0.7	13.2 ± 1.0	–2.2 ± 1.0	2.3 ± 0.2
Pooled	13.4 ± 1.5	6.9 ± 0.9	6.2 ± 1.4	–1.8 ± 1.8	2.2 ± 0.2
^***a***^Estimates are obtained from a multivariate linear regression model having dust advection indicator and dummies for months as predictors. ^***b***^Days with desert dust are defined as days with desert dust advection and estimated PM_10_ concentrations at ground level > 0 μg/m^3^. ^***c***^Ordered by latitude, north to south. ^***d***^Emilia-Romagna includes the cities of Parma, Reggio Emilia, and Modena.					

*Association of exposures with mortality/hospitalizations.* The mean daily counts of total natural mortality ranged from 11 in Bologna to 81 in Athens, and mean numbers of combined cardiovascular and respiratory admissions among those ≥ 15 years of age ranged from 24 in Palermo to 189 in Madrid (see Supplemental Material, Table S3). Three cities (Marseille, Athens, and Thessaloniki) contributed only to the mortality analysis. All cities contributed with at least 3 complete years of daily observations.

**Figure 1:**
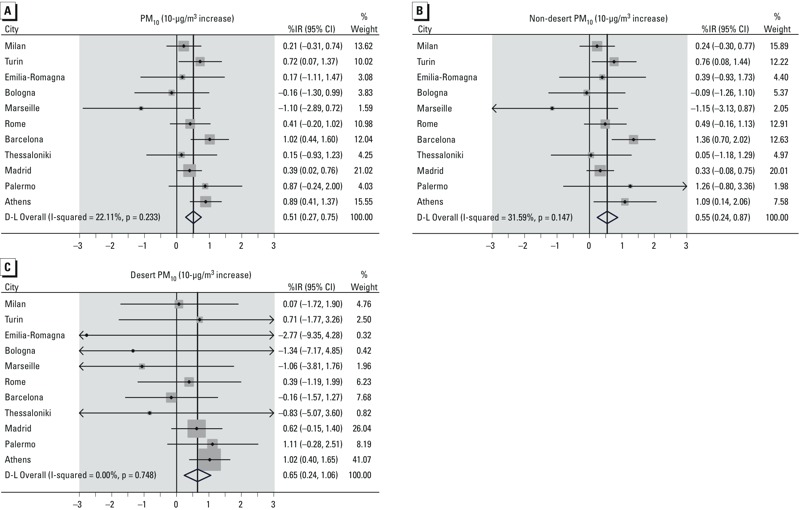
Forest plot with results on estimated percent increases (95% CI) in risk of natural mortality associated with 10-μg/m^3^ increase in total PM_10_ (*A*), non-desert PM_10_ (*B*), and desert PM_10_ (*C*).****Points represent city-specific association estimates, with corresponding 95% CIs (bars). The shaded boxes represent the weights attributed to each estimate in the meta-analysis. Finally, the diamond in the bottom part represents the meta-analytical effect estimate. D-L, DerSimonian and Laird method.

Table 3 shows the associations between total PM_10_ (from single-pollutant models) and source-specific PM_10_ (from two-pollutant models) with daily mortality and hospital admissions. Total PM_10_ was associated with all the study outcomes: Increments of 10 μg/m^3^ were associated with 0.51% (lag 0–1, 95% CI: 0.27, 0.75%), 0.66% (lag 0–5, 95% CI: –0.02, 1.34%), and 2.01% (lag 0–5, 95% CI: 0.92, 3.12%) increases in natural, cardiovascular, and respiratory mortality, respectively. Similar results were found for cardiorespiratory admissions. Associations of mortality and hospitalizations with 10-μg/m^3^ increases of desert and non-desert PM_10_ were similar for all natural mortality (0.65%; 95% CI: 0.24, 1.06 and 0.55%; 95% CI: 0.24, 0.87, respectively), though the association with desert dust appeared stronger for cardiovascular mortality (1.10%; 95% CI: 0.16, 2.06 compared with 0.49%; 95% CI: –0.31, 1.29 for non-desert dust) and weaker for respiratory mortality (1.28%; 95% CI: –0.42, 3.01 compared with 2.46%; 95% CI: 0.96, 3.98). In general, city-specific estimates of the association with desert PM_10_ were homogeneous (*p*-value of heterogeneity > 0.05), whereas consistent heterogeneity was found for the association of non-desert PM_10_ with cardiorespiratory mortality and cardiovascular admissions (*p*-value < 0.05) (Table 3 and Figure 1). Within-city differences between associations of natural mortality with desert and non-desert PM_10_ were not significant (pooled *p*-value 0.72) except in Barcelona, where the association was stronger for non-desert PM_10_ (1.36%; 95% CI: 0.70, 2.03) than desert PM_10_ (–0.16%; 95% CI: –1.57, 1.27) (*p* = 0.05).

**Table 3:** Estimated percent increase (95% CI) in risk of mortality and hospital admissions associated with 10-μg/m^3^ increase in non-desert and desert PM_10_.*^a^*

Outcome	Lag days	PM_10_			Non-desert PM_10_			Desert PM_10_		
		% IR (95% CI)	*I*^2^	Het *p*‑value	% IR (95% CI)	*I*^2^	Het *p*‑value	% IR (95% CI)	*I*^2^	Het *p*‑value
Mortality
Natural	0–1	0.51 (0.27, 0.75)	22	0.23	0.55 (0.24, 0.87)	32	0.15	0.65 (0.24, 1.06)	0	0.75
Cardiovascular	0–5	0.66 (–0.02, 1.34)	40	0.08	0.49 (–0.31, 1.29)	46	0.04	1.10 (0.16, 2.06)	0	0.77
Respiratory	0–5	2.01 (0.92, 3.12)	31	0.15	2.46 (0.96, 3.98)	41	0.07	1.28 (–0.42, 3.01)	0	1.00
Hospital admissions
Cardiovascular, age ≥ 15	0–1	0.29 (0.00, 0.58)	41	0.10	0.37 (–0.04, 0.78)	59	0.02	0.32 (–0.24, 0.89)	0	0.50
Respiratory, age ≥ 15	0–5	0.69 (0.20, 1.19)	32	0.17	0.62 (0.03, 1.21)	21	0.27	0.70 (–0.45, 1.87)	10	0.35
Respiratory, age 0–14	0–5	1.66 (0.93, 2.39)	0	0.47	1.82 (0.77, 2.88)	24	0.23	2.47 (0.22, 4.77)	9	0.36
*I*^2^ statistics represents the amount (%) of heterogeneity among city-specific estimates; Heterogeneity (Het) *p*-value is calculated from the χ^2^ test on the Cochran’s *Q* statistic. ^***a***^The estimates for non-desert and desert PM_10_ are obtained from two-pollutant models adjusted for the other PM source in turn, whereas the estimates for PM_10_ are from single-pollutant models.										

We tested whether desert dust days were associated with increased mortality and hospitalizations as an independent risk factor. When a dichotomous exposure variable (desert dust, yes/no) was evaluated, we found a weak evidence of dust being a risk factor per se, with % increases of mortality on dust versus no-dust days equal to 0.94% (95% CI: 0.07, 1.81%), 0.67% (95% CI: –2.38, 3.82%), and 0.21% (95% CI: –3.50, 4.06%) for natural, cardiovascular, and respiratory causes, respectively. Estimates for admissions were 0.47% (95% CI: –0.50, 1.46%), 0.85% (95% CI: –0.75, 2.47%), and 2.66% (95% CI: –0.19, 5.59%) for cardiovascular (age ≥ 15 years), respiratory (age ≥ 15 years), and respiratory (age 0–14 years) diseases, respectively (data not shown). We also evaluated desert days as an effect modifier of the PM_10_–mortality association using an interaction term. We obtained similar associations between total PM_10_ and natural mortality on dust and no-dust days, with estimates, per 10 μg/m^3^, respectively of 0.74% (95% CI: 0.25, 1.23%) and 0.48% (95% CI: 0.18, 0.78%). A similar result was obtained when we evaluated dust advection as an effect modifier of the non-desert PM_10_–mortality association (data not shown).

**Figure 2:**
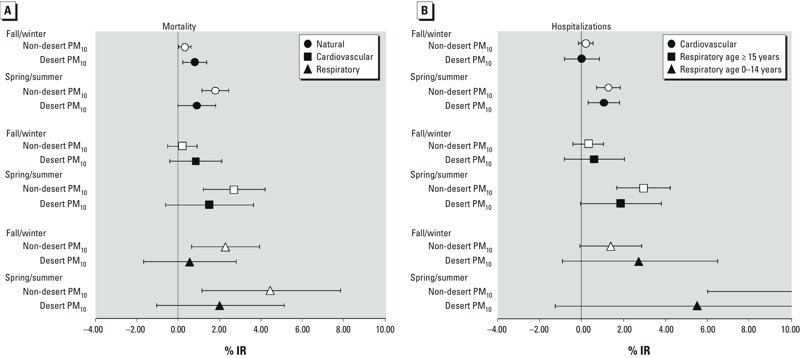
Estimated percent increase (95% CI) in risk of mortality (*A*) and hospitalizations (*B*) associated with 10-μg/m^3^ increase in non-desert (white symbols) and desert (black symbols) PM_10_, by season.

Results of the effect modification by season are reported in Figure 2. As expected, associations with 10-μg/m^3^ increases in both PM_10_ sources were stronger during the warmer season. However, associations with PM_10_ during the spring and summer months were always stronger for non-desert PM_10_ than desert PM_10_. Associations with respiratory admissions during warmer months were the strongest for both exposures, but also were imprecise (12.4%; 95% CI: 6.00, 19.18% and 5.51%; 95% CI: –1.29, 12.78% for 10-μg/m^3^ increases in non-desert and desert PM_10_, respectively).

Finally, the pooled concentration–response functions between non-desert and desert PM_10_ with natural mortality are displayed in Supplemental Material (Figure S2). We found increasing risks of mortality for non-desert PM_10_ concentrations rising up to 60 μg/m^3^, with a flattening of the relationship for higher values of PM_10_. In contrast, the relationship with desert PM_10_ was linear, though the estimates at higher concentrations were affected by large statistical uncertainty. For each city, we have compared the spline and the linear model with likelihood-ratio tests: Most of the cities were consistent with a linear association between natural mortality and desert PM_10_, and a few of them were consistent with a nonlinear relationship with non-desert PM_10_ (see Supplemental Material, Table S4).

## Discussion

To our knowledge, this is the first study investigating the association between PM_10_ originating from desert and non-desert sources with health outcomes in multiple European locations of the Mediterranean area. We estimated statistically significant associations of both PM_10_ sources with mortality and hospital admissions, with no major differences in the magnitude of effects both overall and within cities. Several studies have been conducted in the last years in Southern Europe evaluating the possible role of desert dust advection as effect modifier in the relationship between PM exposure and mortality. The first study from Pérez et al. (2008) reported higher association of the coarse PM with natural mortality on dust days than no-dust days in Barcelona, though no differences were found for fine PM (Pérez et al. 2008). A few years later the same authors evaluated cause-specific mortality, finding evidence of an effect modification induced by dust advection on cardiovascular mortality only (Pérez et al. 2012b), thus confirming the results previously reported in Rome (Mallone et al. 2011) and Madrid (Tobías et al. 2011), and later replicated in Nicosia, Cyprus (Neophytou et al. 2013). On the other hand, two studies conducted in Athens (Samoli et al. 2011a) and Emilia-Romagna (Zauli Sajani et al. 2011) showed desert dust outbreaks as being an independent risk factor for mortality, but the authors questioned its potential role as modifier of the PM–mortality association because they estimated similar associations in dust and no-dust days (Zauli Sajani et al. 2011) or statistically significant associations with PM on no-dust days only (Samoli et al. 2011a). The same inconsistencies emerge from the few studies conducted in Southern Europe on morbidity end points (Alessandrini et al. 2013; Middleton et al. 2008; Reyes et al. 2014; Samoli et al. 2011b).

The present study was designed to compare the associations of the sources of PM_10_, desert and non-desert, with mortality and hospitalizations. In this regard, it is not directly comparable with the aforementioned studies because size-specific PM associations were not reported and the effect modification was only marginally investigated as a secondary analysis. In contrast, in the primary approach—two-exposure models with no interaction terms—no formal effect modification hypothesis was tested. Nonetheless, we reached the same conclusion that desert dust advections represent a threat for human health, and that the health effects of dust-derived PM_10_ are of the same (or similar) magnitude as those reported for anthropogenic sources of air pollution. Specifically, we also compared the estimates of association between non-desert and desert PM_10_ with natural mortality in each city and overall, and found no evidence of heterogeneity in the two estimates in most of the cities. The only other study that applied a similar design was conducted in Barcelona (Pérez et al. 2012a), and reported stronger associations with PM_10_ during dust days compared with no-dust days, especially for the fraction of PM_10_ not originated from desert sources. When we applied the effect modification analysis on either total PM_10_ or non-desert PM_10_ with a desert dust indicator (yes/no), we obtained results similar to those found in other studies: similar associations with PM_10_ on dust and no-dust days.

The plausibility of health effects due to desert PM exposure is supported by several studies. Numerous species of fungi, bacteria, and viruses have been found on many dust samples, and some authors have speculated that the transport of microorganisms, especially over water bodies, would be favored by the tolerable humidity levels and attenuation of ultraviolet light by the particle load of dust clouds (Griffin 2007). Furthermore, the toxicity of desert dust might be enhanced by the mixing with anthropogenic sources of air pollution during cloud formation or capture in downwind transport (e.g., adsorption of pesticides, industrial emissions) (Rodríguez et al. 2001). Similarly, specific desert dust episodes are associated with a lowering of the mixing layer height, thus enhancing local and regional atmospheric pollution (and increasing fine as well as coarse PM) from all sources and consequently increasing mortality. This hypothesis has been recently confirmed for Barcelona by Pandolfi et al. (2014).

Different source areas and patterns of transport to western and eastern Mediterranean areas could also be responsible for different toxicological properties of desert dust, and therefore may partially explain the inconsistencies in previous epidemiological results. Though the Arabian Peninsula is a large source of atmospheric dust overall, such particles have an influence only in the extreme east of the Mediterranean. Our previous article on dust exposure (Pey et al. 2013) revealed an impact from this region only in Cyprus but not westward. Therefore, we have assumed that all the cities included in this study were affected only by Saharan dust advection episodes. Middleton and Goudie (2001) identified South Algeria and West Sahara as the major source areas for dust transport in the western Mediterranean basin. In contrast, the eastern Mediterranean would be more affected by dust transported by air masses from Libya and Egypt (Kallos et al. 2006). The different regions of the Sahara desert have been shown to have distinct mineralogical properties (Moreno et al. 2006), likely affecting their toxicological potential. Also, dust transport over the western and eastern sides of the Mediterranean is characterized by different mechanisms. African dust episodes over the western and central basin are very frequent in spring/summer, although moderate in intensity due to the intricate transport processes (dust travels at very high altitudes) (Karanasiou et al. 2012). In contrast, desert dust transport in the eastern basin is typically induced by cyclones moving eastward across the Mediterranean and North Africa, transporting dust at surface levels (Pey et al. 2013). These flows may be enhanced under specific scenarios (Moulin et al. 1998), giving rise to short but intense dust episodes.

Associations with desert and non-desert PM_10_ were stronger during the warmer months, as previously reported (Samoli et al. 2013; Stafoggia et al. 2013). We also found slightly weaker associations with desert PM_10_ than non-desert PM_10_ during the warmer season. This could have had an impact on the overall estimates due to the higher frequency of dust outbreaks in spring and summer. However, it is possible that higher air conditioning use during desert dust days could have reduced effect estimates for desert PM_10_. A limitation of our approach has been to classify seasons into two broad 6-month time windows, though we have shown that dust occurrence can be different in spring and summer months. However, it was not possible to use a finer classification of season in the epidemiological analysis of effect modification due to power constraints.

The main strength of the study has been the application of a standardized protocol for desert dust detection and quantification, coupled with health data collection and epidemiological investigation in multiple cities of the Euro-Mediterranean area. Advanced modeling outputs and satellite images have been applied on a large spatiotemporal scale covering four countries and 10 years of daily observations. Second, we were able to separate different sources of particulate matter by applying official EU methodologies, and thus could disentangle the independent short-term health effects of desert and non-desert PM_10_. Third, we evaluated both mortality and hospital admissions as study outcomes, thus providing valuable information on the potential mechanisms underlying the reported health effects. Major limitations include the lack of experimental measurements of mineral dust during desert dust advections, and the lack of PM speciation data characterizing the composition of particulate matter during dust and no-dust days. Furthermore, we were unable to quantify the desert contribution to daily fine and coarse PM concentrations because few data were available on PM_2.5_ and PM_2.5–10_ at rural background monitoring sites. Our statistics on desert dust days are not equal to those of previous reports (Pey et al. 2013; Querol et al. 2009b) because we defined a desert dust day as when *a*) desert advection was identified through operational models and *b*) desert PM_10_ estimated concentration was > 0 μg/m^3^. Finally, we have used PM concentrations from fixed ambient monitors to infer individual exposures: This might have induced exposure misclassification with Berkson-type error, with possible loss of precision but no induced bias on the regression estimates.

## Conclusions

Our results provide consistent evidence that desert dust outbreaks are an important risk factor to human health, and that exposure to PM originated by such natural events is not harmless. In particular, we estimated excess mortality and hospitalizations associated with desert PM_10_ of the same magnitude as those reported for non-desert PM_10_.

One of the consequences of climate change is the acceleration of desertification processes in arid and semi-arid regions, which implies an increase in dust outbreaks in the near future (IPCC 2007). Evidence of adverse health effects of both desert and non-desert sources strengthens the need to control for anthropogenic sources, especially on days when desert dust levels are high. Therefore, steps should be undertaken at local and regional levels to ensure public health protection, by reducing anthropogenic emissions and population exposure especially on desert dust advection days.

## Supplemental Material

(632 KB) PDF

## References

[r1] Alessandrini ER, Stafoggia M, Faustini A, Gobbi GP, Forastiere F (2013). Saharan dust and the association between particulate matter and daily hospitalisations in Rome, Italy.. Occup Environ Med.

[r2] Brunekreef B, Forsberg B (2005). Epidemiological evidence of effects of coarse airborne particles on health.. Eur Respir J.

[r3] Escudero M Castillo S Querol X Avila A Alarcón M Viana MM 2005 Wet and dry African dust episodes over eastern Spain. J Geophys Res 110 D18S08; doi:10.1029/2004JD004731

[r4] Escudero M, Querol X, Pey J, Alastuey A, Pérez N, Ferreira F (2007). A methodology for the quantification of the net African dust load in air quality monitoring networks.. Atmos Environ.

[r5] Gkikas A, Hatzianastassiou N, Mihalopoulos N, Katsoulis V, Kazadzis S, Pey J (2013). The regime of desert dust episodes in the Mediterranean based on contemporary satellite observations and ground measurements.. Atmos Chem Phys.

[r6] Griffin DW (2007). Atmospheric movement of microorganisms in clouds of desert dust and implications for human health.. Clin Microbiol Rev.

[r7] Higgins JP, Thompson SG (2002). Quantifying heterogeneity in a meta-analysis.. Stat Med.

[r8] IPCC (Intergovernmental Panel on Climate Change) (2007). Climate Change 2007: the Physical Science Basis: Contribution of Working Group I to the Fourth Assessment Report of the Intergovernmental Panel on Climate Change (Solomon S, Qin D, Manning M, Chen Z, Marquis M, Averyt KB, et al., eds)..

[r9] Jackson D, White IR, Thompson SG (2010). Extending DerSimonian and Laird’s methodology to perform multivariate random effects meta-analyses.. Stat Med.

[r10] Kalivitis N Gerasopoulos E Vrekoussis M Kouvarakis G Kubilay N Hatzianastassiou N 2007 Dust transport over the eastern Mediterranean derived from Total Ozone Mapping Spectrometer, Aerosol Robotic Network, and surface measurements. J Geophys Res 112 D03202; doi:10.1029/2006JD007510

[r11] Kallos G Papadopoulos A Katsafados P Nickovic S 2006 Transatlantic Saharan dust transport: model simulation and results. J Geophys Res 111 D09204; doi:10.1029/2005JD006207

[r12] Karanasiou A, Moreno N, Moreno T, Viana M, de Leeuw F, Querol X (2012). Health effects from Sahara dust episodes in Europe: literature review and research gaps.. Environ Int.

[r13] Katsouyanni K, Samet JM, Anderson HR, Atkinson R, Le Tertre A, Medina S, et al (2009). Air pollution and Health: a North American and European Approach (APHENA). Research Report 142.. http://pubs.healtheffects.org/getfile.php?u=518.

[r14] Levy D, Lumley T, Sheppard L, Kaufman J, Checkoway H (2001). Referent selection in case-crossover analyses of acute health effects of air pollution.. Epidemiology.

[r15] Lu Y, Zeger SL (2007). On the equivalence of case-crossover and time series methods in environmental epidemiology.. Biostatistics.

[r16] Maclure M (1991). The case-crossover design: a method for studying transient effects on the risk of acute events.. Am J Epidemiol.

[r17] Mallone S Stafoggia M Faustini A Gobbi GP Marconi A Forastiere F 2011 Saharan dust and associations between particulate matter and daily mortality in Rome, Italy. Environ Health Perspect 119 1409 1414; doi:10.12989/ehp.1003026 21970945 PMC3230430

[r18] Middleton NJ, Goudie AS (2001). Saharan dust: sources and trajectories.. Trans Inst Br Geogr.

[r19] Middleton N Yiallouros P Kleanthous S Kolokotroni O Schwartz J Dockery DW 2008 A 10-year time-series analysis of respiratory and cardiovascular morbidity in Nicosia, Cyprus: the effect of short-term changes in air pollution and dust storms. Environ Health 7 39; doi:10.1186/1476-069X-7-39 18647382 PMC2517071

[r20] Moreno T, Querol X, Castillo S, Alastuey A, Cuevas E, Herrmann L (2006). Geochemical variations in aeolian mineral particles from the Sahara–Sahel Dust Corridor.. Chemosphere.

[r21] Moulin C, Lambert CE, Dayan U, Masson V, Ramonet M, Bousquet P (1998). Satellite climatology of African dust transport in the Mediterranean atmosphere.. J Geophys Res.

[r22] Neophytou AM, Yiallouros P, Coull BA, Kleanthous S, Pavlou P, Pashiardis S (2013). Particulate matter concentrations during desert dust outbreaks and daily mortality in Nicosia, Cyprus.. J Expo Sci Environ Epidemiol.

[r23] Pandolfi M, Tobias A, Alastuey A, Sunyer J, Schwartz J, Lorente J (2014). Effect of atmospheric mixing layer depth variations on urban air quality and daily mortality during Saharan dust outbreaks.. Sci Total Environ.

[r24] Pérez L, Tobías A, Pey J, Pérez N, Alastuey A, Sunyer J (2012a). Effects of local and Saharan particles on cardiovascular disease mortality.. Epidemiology.

[r25] Pérez L, Tobias A, Querol X, Künzli N, Pey J, Alastuey A (2008). Coarse particles from Saharan dust and daily mortality.. Epidemiology.

[r26] Pérez L, Tobías A, Querol X, Pey J, Alastuey A, Díaz J (2012b). Saharan dust, particulate matter and cause-specific mortality: a case-crossover study in Barcelona (Spain).. Environ Int.

[r27] Perrino C, Canepari S, Catrambone M, Dalla Torre S, Rantica E, Sargolini T (2009). Influence of natural events on the concentration and composition of atmospheric particulate matter.. Atmos Environ.

[r28] Pey J, Querol X, Alastuey A, Forastiere F, Stafoggia M (2013). African dust outbreaks over the Mediterranean Basin during 2001–2011: PM_10_ concentrations, phenomenology and trends, and its relation with synoptic and mesoscale meteorology.. Atmos Chem Phys.

[r29] Pope CA, Dockery DW (2006). Health effects of fine particulate air pollution: lines that connect.. J Air Waste Manag Assoc.

[r30] Prospero JM, Ginoux P, Torres O, Nicholson SE, Gill TE (2002).

[r31] Querol X, Alastuey A, Pey J, Cusack M, Pérez N, Mihalopoulos N (2009a). Variability in regional background aerosols within the Mediterranean.. Atmos Chem Phys.

[r32] Querol X, Pey J, Pandolfi M, Alastuey A, Cusack M, Moreno T (2009b). African dust contributions to mean ambient PM_10_ mass-levels across the Mediterranean Basin.. Atmos Environ.

[r33] R Core Team (2011). R: A Language and Environment for Statistical Computing.. http://www.R-project.org.

[r34] Reyes M, Díaz J, Tobias A, Montero JC, Linares C (2014). Impact of Saharan dust particles on hospital admissions in Madrid (Spain).. Int J Environ Health Res.

[r35] Rodríguez S, Querol X, Alastuey A, Kallos G, Kakaliagou O (2001). Saharan dust contributions to PM_10_ and TSP levels in Southern and Eastern Spain.. Atmos Environ.

[r36] Salvador P, Alonso-Pérez S, Pey J, Artíñano B, de Bustos JJ, Alastuey A (2014). African dust outbreaks over the western Mediterranean Basin: 11-year characterization of atmospheric circulation patterns and dust source areas.. Atmos Chem Phys.

[r37] Samoli E, Kougea E, Kassomenos P, Analitis A, Katsouyanni K (2011a). Does the presence of desert dust modify the effect of PM_10_ on mortality in Athens, Greece?. Sci Total Environ.

[r38] Samoli E, Nastos PT, Paliatsos AG, Katsouyanni K, Priftis KN (2011b). Acute effects of air pollution on pediatric asthma exacerbation: evidence of association and effect modification.. Environ Res.

[r39] Samoli E, Stafoggia M, Rodopoulou S, Ostro B, Alessandrini E, Basagaña X (2014). Which specific causes of death are associated with short term exposure to fine and coarse particles in Southern Europe? Results from the MED-PARTICLES project.. Environ Int.

[r40] Samoli E Stafoggia M Rodopoulou S Ostro B Declercq C Alessandrini E 2013 Associations between fine and coarse particles and mortality in Mediterranean cities: results from the MED-PARTICLES project. Environ Health Perspect 121 932 938; doi:10.1289/ehp.1206124 23687008 PMC3734494

[r41] Schwartz J, Zanobetti A (2000). Using meta-smoothing to estimate dose-response trends across multiple studies, with application to air pollution and daily death.. Epidemiology.

[r42] Stafoggia M Samoli E Alessandrini E Cadum E Ostro B Berti G 2013 Short-term associations between fine and coarse particulate matter and hospitalizations in Southern Europe: results from the MED-PARTICLES project. Environ Health Perspect 121 1026 1033; doi:10.1289/ehp.1206151 23777832 PMC3764077

[r43] Tobías A, Pérez L, Díaz J, Linares C, Pey J, Alastuey A (2011). Short-term effects of particulate matter on total mortality during Saharan dust outbreaks: a case-crossover analysis in Madrid (Spain).. Sci Total Environ.

[r44] Zauli Sajani S, Miglio R, Bonasoni P, Cristofanelli P, Marinoni A, Sartini C (2011). Saharan dust and daily mortality in Emilia-Romagna (Italy).. Occup Environ Med.

