# Supplementary material for: Desert Dust Outbreaks in Southern Europe: Contribution to Daily PM10 Concentrations and Short-Term Associations with Mortality and Hospital Admissions
Source: Environ Health Perspect. 2015 Jul 24;124(4):413–9. doi: 10.1289/ehp.1409164 (PMC4829979; doi:10.1289/ehp.1409164)
Supplement: (632 KB) PDF [file ehp.1409164.s001.acco.pdf]

*Environ Health Perspect*
DOI: 10.1289/ehp.1409164

**Note to Readers:** *EHP* strives to ensure that all journal content is accessible to all readers. However, some figures and Supplemental Material published in *EHP* articles may not conform to 508 standards due to the complexity of the information being presented. If you need assistance accessing journal content, please contact ehp508@niehs.nih.gov. Our staff will work with you to assess and meet your accessibility needs within 3 working days.

# Supplemental Material

# Desert Dust Outbreaks in Southern Europe: Contribution to Daily $PM_{10}$ Concentrations and Short-Term Associations with Mortality and Hospital Admissions

Massimo Stafoggia, Stefano Zauli-Sajani, Jorge Pey, Evangelia Samoli, Ester Alessandrini, Xavier Basagaña, Achille Cernigliaro, Monica Chiusolo, Moreno Demaria, Julio Díaz, Annunziata Faustini, Klea Katsouyanni, Apostolos G. Kelessis, Cristina Linares, Stefano Marchesi, Sylvia Medina, Paolo Pandolfi, Noemí Pérez, Xavier Querol, Giorgia Randi, Andrea Ranzi, Aurelio Tobias, Francesco Forastiere, and the MED-PARTICLES Study Group

## Table of contents

**Figure S1.** Relative frequencies of desert dust days in the eleven cities of the MED-PARTICLES project, by season. Cities ordered by latitude, North to South. Desert dust days are defined as days with desert dust advection and estimated $PM_{10}$ concentrations at ground level > 0 μg/m$^3$

**Table S1.** Daily PM concentrations (mean ± SD, μg/m$^3$) by desert dust advection, in the eleven cities of the MED-PARTICLES project

**Table S2.** City-specific Pearson correlation coefficients between environmental variables. The lower triangle includes the correlations on days with desert dust. The upper triangle includes the correlations on days without desert dust. The cities are ordered by latitude, North to South. PM and ozone are measured as μg/m$^3$, air temperature as °C

**Table S2.** City-specific Pearson correlation coefficients between environmental variables. The lower triangle includes the correlations on days with desert dust. The upper triangle includes the

correlations on days without desert dust. The cities are ordered by latitude, North to South. PM and ozone are measured as μg/m$^3$, air temperature as °C (continued)

**Table S2.** City-specific Pearson correlation coefficients between environmental variables. The lower triangle includes the correlations on days with desert dust. The upper triangle includes the correlations on days without desert dust. The cities are ordered by latitude, North to South. PM and ozone are measured as μg/m$^3$, air temperature as °C (continued)

**Table S3.** Daily mean counts of cause-specific mortality and hospital admissions in the eleven cities of the MED-PARTICLES project

**Figure S2.** Concentration–response relationship between non-desert $PM_{10}$ (A) and desert $PM_{10}$ (B) with natural mortality, lag 0-1. Values are estimated percent increases (95% CI) of total natural mortality associated with increases of $PM_{10}$ levels relative to 5 μg/m$^3$

**Table S4.** P-values of the city-specific likelihood-ratio tests comparing the models with natural spline and linear term for non-desert $PM_{10}$ and desert $PM_{10}$ exposures versus natural mortality

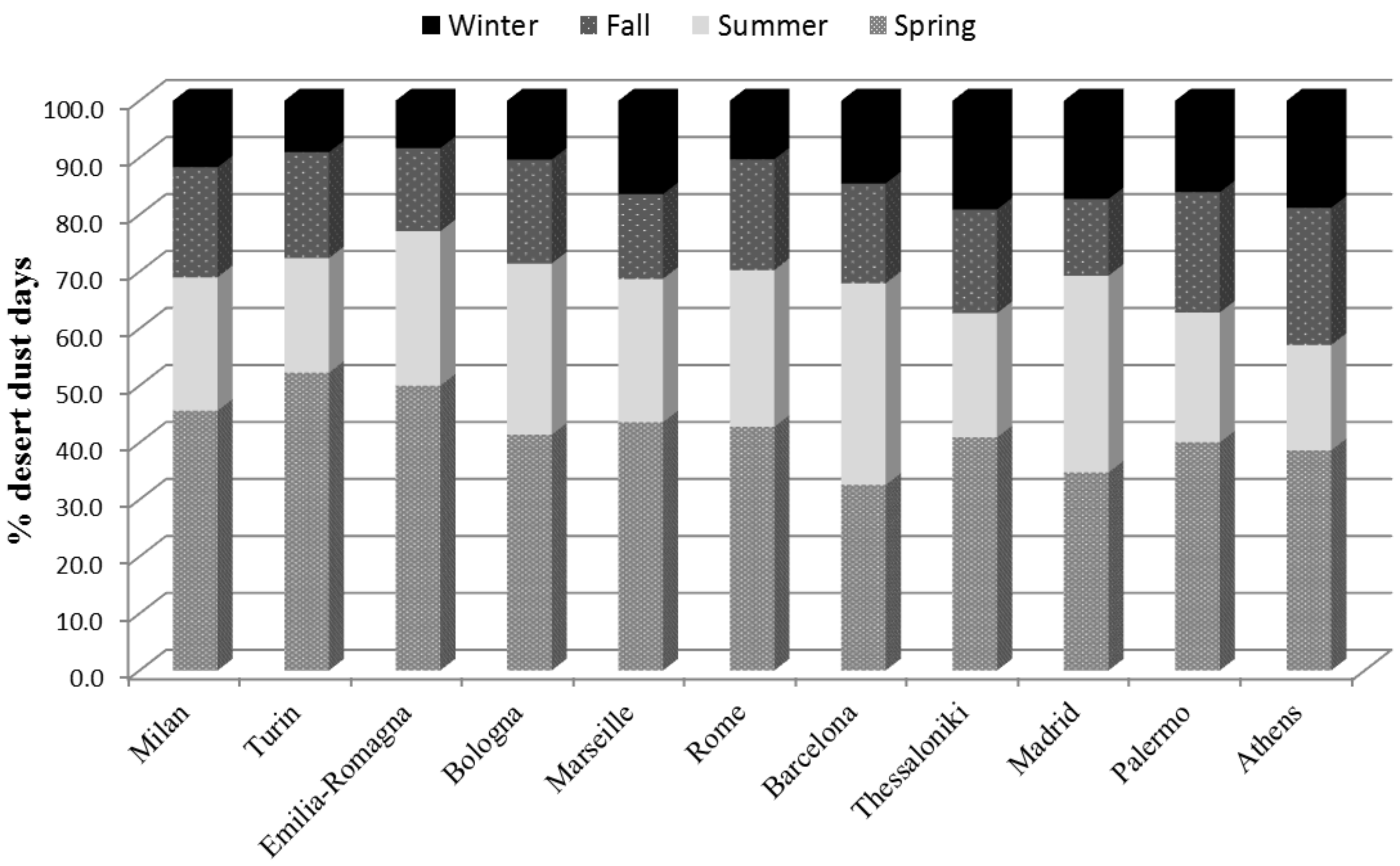


**Figure S1.** Relative frequencies of desert dust days in the eleven cities of the MED-PARTICLES project, by season. Cities ordered by latitude, North to South. Desert dust days are defined as days with desert dust advection and estimated $PM_{10}$ concentrations at ground level > 0 μg/m$^3$

**Table S1.** Daily PM concentrations (mean ± SD, μg/m$^3$) by desert dust advection, in the eleven cities of the MED-PARTICLES project

| City[a] | Study period | Days with no desert dust[b] | | | Days with desert dust[b] | | | | |
|---|---|---|---|---|---|---|---|---|---|
| | | $PM_{10}$ | $PM_{2.5}$ | $PM_{2.5-10}$ | $PM_{10}$ | Non-desert $PM_{10}$ | Desert $PM_{10}$ | $PM_{2.5}$ | $PM_{2.5-10}$ |
| Milan | 2007-2010 | 44.7 ± 31.5 | 31.4 ± 26.3 | 14.2 ± 9.6 | 45.1 ± 23.9 | 28.2 ± 16.9 | 16.9 ± 15.1 | 28.2 ± 19.1 | 16.3 ± 8.8 |
| Turin | 2006-2010 | 48.5 ± 37.1 | 34.8 ± 28.9 | - | 47.0 ± 29.7 | 30.9 ± 20.4 | 16.0 ± 15.9 | 32.6 ± 23.3 | - |
| Emilia-Romagna[c] | 2008-2010 | 33.9 ± 19.4 | 21.6 ± 15.2 | 12.1 ± 6.3 | 38.1 ± 18.0 | 29.0 ± 16.0 | 9.0 ± 8.8 | 21.0 ± 11.7 | 17.0 ± 8.3 |
| Bologna | 2006-2010 | 38.0 ± 21.7 | 25.8 ± 18.9 | - | 42.9 ± 21.8 | 32.3 ± 21.4 | 10.3 ± 9.7 | 27.1 ± 16.7 | - |
| Marseille | 2006-2008 | 24.1 ± 9.3 | 15.8 ± 9.5 | 7.5 ± 5.9 | 33.1 ± 12.8 | 20.5 ± 10.9 | 12.5 ± 10.7 | 19.1 ± 8.3 | 11.9 ± 8.8 |
| Rome | 2005-2010 | 33.7 ± 14.0 | 19.5 ± 10.4 | 12.2 ± 5.9 | 39.7 ± 13.5 | 28.5 ± 10.3 | 11.2 ± 10.1 | 20.0 ± 7.7 | 17.9 ± 8.9 |
| Barcelona | 2003-2010 | 34.4 ± 15.4 | 22.4 ± 10.6 | 12.0 ± 9.3 | 48.0 ± 18.8 | 35.3 ± 17.8 | 12.9 ± 11.9 | 31.2 ± 13.1 | 17.0 ± 12.4 |
| Thessaloniki | 2007-2009 | 46.6 ± 19.7 | 28.4 ± 11.2 | 17.5 ± 10.0 | 53.9 ± 16.4 | 42.1 ± 13.7 | 11.8 ± 11.3 | 33.8 ± 9.6 | 20.1 ± 8.4 |
| Madrid | 2001-2009 | 32.4 ± 17.8 | 15.9 ± 8.8 | 15.9 ± 10.7 | 52.1 ± 21.7 | 33.6 ± 20.0 | 19.0 ± 18.4 | 24.8 ± 11.8 | 25.7 ± 15.4 |
| Palermo | 2006-2009 | 30.5 ± 9.9 | - | - | 47.0 ± 23.0 | 30.5 ± 10.6 | 16.5 ± 21.3 | - | - |
| Athens | 2007-2009 | 32.3 ± 12.3 | 20.7 ± 8.5 | 11.4 ± 7.2 | 54.0 ± 28.8 | 34.0 ± 7.9 | 20.0 ± 26.3 | 29.0 ± 12.4 | 24.8 ± 23.2 |

[a] Ordered by latitude, North to South

[b] Desert dust days are defined as days with desert dust advection and estimated $PM_{10}$ concentrations at ground level > 0 μg/m$^3$

[c] Emilia Romagna includes the cities of Parma, Reggio Emilia, and Modena

**Table S2.** City-specific Pearson correlation coefficients between environmental variables. The lower triangle includes the correlations on days with desert dust. The upper triangle includes the correlations on days without desert dust. The cities are ordered by latitude, North to South. PM and ozone are measured as μg/m$^3$, air temperature as °C

| Milan | Non-desert $PM_{10}$ | Desert $PM_{10}$ | $PM_{2.5}$ | $PM_{2.5-10}$ | Ozone | Air temperature |
|---|---|---|---|---|---|---|
| Non-desert $PM_{10}$ | | - | 0.96 | 0.68 | -0.50 | -0.52 |
| Desert $PM_{10}$ | 0.11 | | - | - | - | - |
| $PM_{2.5}$ | 0.62 | 0.77 | | 0.45 | -0.55 | -0.58 |
| $PM_{2.5-10}$ | 0.59 | 0.31 | 0.25 | | -0.12 | -0.13 |
| Ozone | -0.34 | -0.43 | -0.63 | -0.01 | | 0.87 |
| Air temperature | -0.35 | -0.40 | -0.64 | 0.03 | 0.86 | |
| **Turin** | | | | | | |
| Non-desert $PM_{10}$ | | - | 0.94 | - | -0.56 | -0.55 |
| Desert $PM_{10}$ | 0.28 | | - | - | - | - |
| $PM_{2.5}$ | 0.69 | 0.75 | | - | -0.60 | -0.59 |
| $PM_{2.5-10}$ | - | - | - | | - | - |
| Ozone | -0.50 | -0.26 | -0.56 | - | | 0.84 |
| Air temperature | -0.45 | -0.32 | -0.58 | - | 0.79 | |
| **Emilia Romagna** | | | | | | |
| Non-desert $PM_{10}$ | | - | 0.96 | 0.73 | -0.45 | -0.44 |
| Desert $PM_{10}$ | -0.04 | | - | - | - | - |
| $PM_{2.5}$ | 0.88 | 0.31 | | 0.52 | -0.49 | -0.53 |
| $PM_{2.5-10}$ | 0.65 | 0.58 | 0.63 | | -0.16 | -0.06 |
| Ozone | -0.41 | 0.07 | -0.43 | -0.10 | | 0.88 |
| Air temperature | -0.42 | 0.16 | -0.44 | 0.00 | 0.83 | |
| **Bologna** | | | | | | |
| Non-desert $PM_{10}$ | | - | 0.96 | - | -0.50 | -0.50 |
| Desert $PM_{10}$ | -0.19 | | - | - | - | - |
| $PM_{2.5}$ | 0.90 | 0.13 | | - | -0.48 | -0.49 |
| $PM_{2.5-10}$ | - | - | - | | - | - |
| Ozone | -0.55 | 0.08 | -0.49 | - | | 0.84 |
| Air temperature | -0.58 | 0.27 | -0.55 | - | 0.77 | |

**Table S2.** City-specific Pearson correlation coefficients between environmental variables. The lower triangle includes the correlations on days with desert dust. The upper triangle includes the correlations on days without desert dust. The cities are ordered by latitude, North to South. PM and ozone are measured as μg/m$^3$, air temperature as °C (continued)

| Marseille | Non-desert $PM_{10}$ | Desert $PM_{10}$ | $PM_{2.5}$ | $PM_{2.5-10}$ | Ozone | Air temperature |
|---|---|---|---|---|---|---|
| Non-desert $PM_{10}$ | | - | 0.80 | 0.25 | -0.10 | -0.08 |
| Desert $PM_{10}$ | -0.29 | | - | - | - | - |
| $PM_{2.5}$ | 0.42 | 0.51 | | -0.38 | -0.33 | -0.37 |
| $PM_{2.5-10}$ | 0.53 | 0.44 | 0.07 | | 0.30 | 0.48 |
| Ozone | 0.11 | -0.13 | -0.07 | 0.12 | | 0.73 |
| Air temperature | 0.25 | 0.00 | -0.18 | 0.52 | 0.59 | |

| Rome | | | | | | |
|---|---|---|---|---|---|---|
| Non-desert $PM_{10}$ | | - | 0.91 | 0.69 | -0.32 | -0.21 |
| Desert $PM_{10}$ | -0.12 | | - | - | - | - |
| $PM_{2.5}$ | 0.63 | 0.40 | | 0.36 | -0.39 | -0.31 |
| $PM_{2.5-10}$ | 0.44 | 0.67 | 0.33 | | -0.01 | 0.14 |
| Ozone | -0.15 | -0.02 | -0.17 | -0.02 | | 0.75 |
| Air temperature | -0.04 | 0.17 | 0.00 | 0.18 | 0.65 | |

| Barcelona | | | | | | |
|---|---|---|---|---|---|---|
| Non-desert $PM_{10}$ | | - | 0.80 | 0.73 | -0.15 | 0.02 |
| Desert $PM_{10}$ | -0.25 | | - | - | - | - |
| $PM_{2.5}$ | 0.50 | 0.45 | | 0.19 | -0.22 | -0.14 |
| $PM_{2.5-10}$ | 0.67 | 0.13 | 0.09 | | 0.01 | 0.19 |
| Ozone | -0.13 | -0.08 | -0.24 | -0.04 | | 0.59 |
| Air temperature | -0.18 | 0.02 | -0.30 | 0.06 | 0.49 | |

| Thessaloniki | | | | | | |
|---|---|---|---|---|---|---|
| Non-desert $PM_{10}$ | | - | 0.95 | 0.92 | -0.33 | -0.17 |
| Desert $PM_{10}$ | -0.15 | | - | - | - | - |
| $PM_{2.5}$ | 0.61 | 0.61 | | 0.77 | -0.21 | -0.07 |
| $PM_{2.5-10}$ | 0.74 | 0.40 | 0.67 | | -0.35 | -0.23 |
| Ozone | -0.55 | 0.12 | -0.27 | -0.45 | | 0.63 |
| Air temperature | -0.30 | 0.21 | -0.04 | -0.16 | 0.70 | |

**Table S2.** City-specific Pearson correlation coefficients between environmental variables. The lower triangle includes the correlations on days with desert dust. The upper triangle includes the correlations on days without desert dust. The cities are ordered by latitude, North to South. PM and ozone are measured as μg/m$^3$, air temperature as °C (continued)

| Madrid | Non-desert $PM_{10}$ | Desert $PM_{10}$ | $PM_{2.5}$ | $PM_{2.5-10}$ | Ozone | Air temperature |
|---|---|---|---|---|---|---|
| Non-desert $PM_{10}$ | | - | 0.90 | 0.94 | -0.35 | 0.03 |
| Desert $PM_{10}$ | -0.36 | | - | - | - | - |
| $PM_{2.5}$ | 0.45 | 0.51 | | 0.70 | -0.42 | 0.04 |
| $PM_{2.5-10}$ | 0.53 | 0.52 | 0.68 | | -0.39 | -0.05 |
| Ozone | -0.34 | -0.06 | -0.23 | -0.28 | | 0.65 |
| Air temperature | -0.12 | 0.09 | 0.11 | -0.03 | 0.70 | |
| **Palermo** | | | | | | |
| Non-desert $PM_{10}$ | | - | - | - | -0.29 | 0.04 |
| Desert $PM_{10}$ | -0.08 | | - | - | - | - |
| $PM_{2.5}$ | - | - | | - | - | - |
| $PM_{2.5-10}$ | - | - | - | | - | - |
| Ozone | -0.21 | -0.15 | - | - | | 0.35 |
| Air temperature | -0.07 | 0.13 | - | - | 0.17 | |
| **Athens** | | | | | | |
| Non-desert $PM_{10}$ | | - | 0.72 | 0.69 | 0.06 | 0.17 |
| Desert $PM_{10}$ | 0.18 | | - | - | - | - |
| $PM_{2.5}$ | 0.62 | 0.50 | | 0.22 | 0.31 | 0.34 |
| $PM_{2.5-10}$ | 0.22 | 0.93 | 0.25 | | -0.17 | -0.06 |
| Ozone | 0.14 | -0.18 | 0.28 | -0.29 | | 0.79 |
| Air temperature | 0.24 | -0.01 | 0.39 | -0.13 | 0.72 | |

**Table S3.** Daily mean counts of cause-specific mortality and hospital admissions in the eleven cities of the MED-PARTICLES project

| City[a] | Study period | Natural mortality | Cardiovascular mortality | Respiratory mortality | Cardiovascular admissions age 15+ | Respiratory admissions age 15+ | Respiratory admissions age 0-14 |
|---|---|---|---|---|---|---|---|
| Milan | 2007-2010 | 35 | 12 | 3 | 39 | 19 | 5 |
| Turin | 2006-2010 | 20 | 8 | 2 | 27 | 12 | 3 |
| Emilia-Romagna[b] | 2008-2010 | 13 | 5 | 1 | 18 | 9 | 1 |
| Bologna | 2006-2010 | 11 | 4 | 1 | 19 | 8 | 1 |
| Marseille | 2006-2008 | 21 | 6 | 1 | - | - | - |
| Rome | 2005-2010 | 58 | 24 | 4 | 81 | 29 | 8 |
| Barcelona | 2003-2010 | 42 | 13 | 5 | 52 | 48 | 6 |
| Thessaloniki | 2007-2009 | 18 | 8 | 2 | - | - | - |
| Madrid | 2001-2009 | 60 | 18 | 9 | 106 | 83 | 24 |
| Palermo | 2006-2009 | 15 | 6 | 1 | 17 | 7 | 3 |
| Athens | 2007-2009 | 81 | 36 | 9 | - | - | - |

[a] Ordered by latitude, North to South

[b] Emilia Romagna includes the cities of Parma, Reggio Emilia, and Modena

(A)

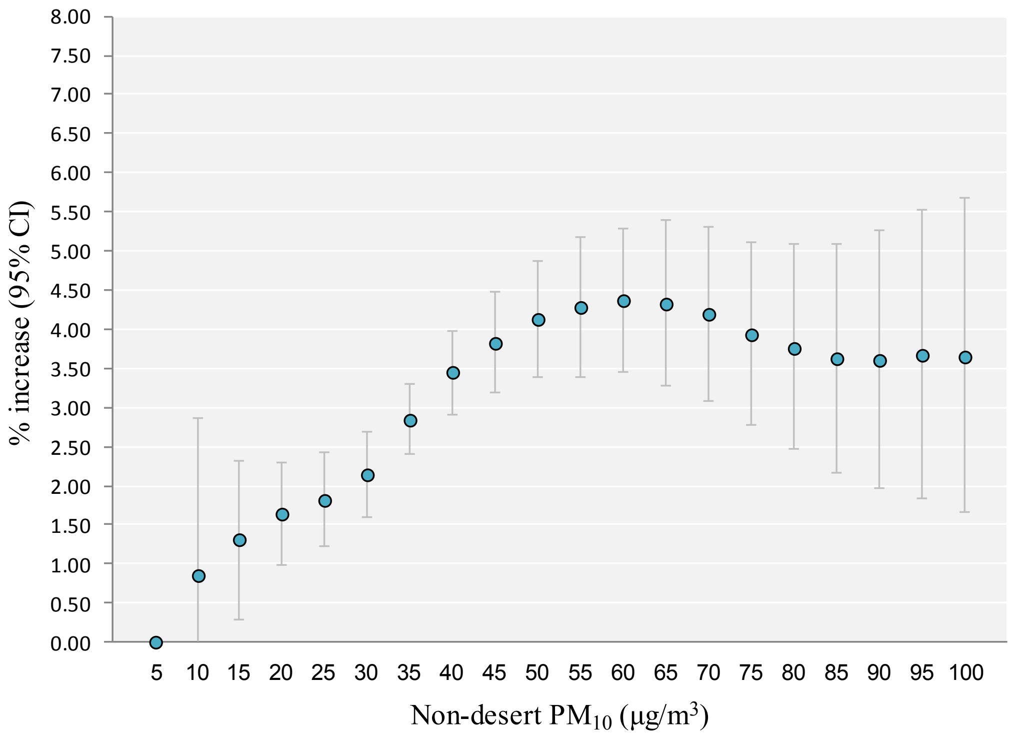


(B)

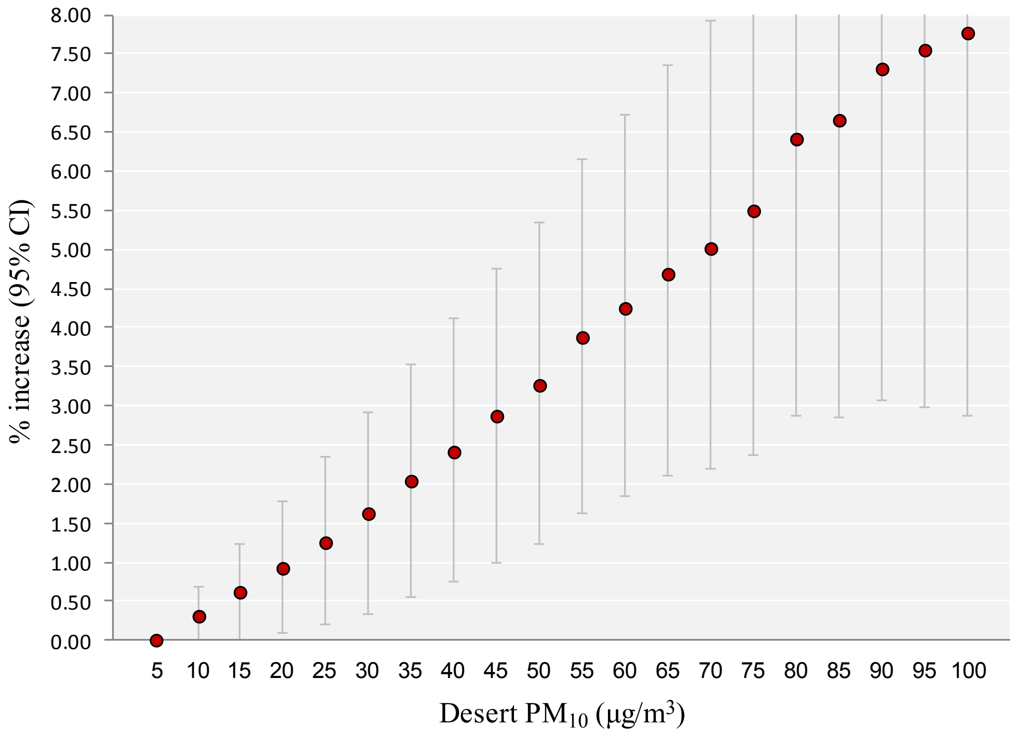


**Figure S2.** Concentration–response relationship between non-desert $PM_{10}$ (A) and desert $PM_{10}$ (B) with natural mortality, lag 0-1. Values are estimated percent increases (95% CI) of total natural mortality associated with increases of $PM_{10}$ levels relative to 5 μg/m$^3$

**Table S4.** P-values of the city-specific likelihood-ratio tests comparing the models with natural spline and linear term for non-desert $PM_{10}$ and desert $PM_{10}$ exposures versus natural mortality

| City | p-value likelihood-ratio test spline model VS linear model | |
|---|---|---|
| | desert $PM_{10}$ | Non-desert $PM_{10}$ |
| Milan | 0.649 | 0.006 |
| Turin | 0.084 | 0.415 |
| Emilia-Romagna | 0.033 | 0.847 |
| Bologna | 0.359 | 0.642 |
| Marseille | 0.277 | 0.205 |
| Rome | 0.323 | 0.312 |
| Barcelona | 0.242 | 0.031 |
| Thessaloniki | 0.803 | 0.631 |
| Madrid | 0.085 | 0.071 |
| Palermo | 0.211 | 0.178 |
| Athens | 0.565 | 0.075 |
